# Mitigation of BMP-induced inflammation in craniofacial bone regeneration and improvement of bone parameters by dietary hesperidin

**DOI:** 10.1038/s41598-024-52566-7

**Published:** 2024-01-31

**Authors:** Patricia A. Miguez, Vinícius de Paiva Gonçalves, Marta L. Musskopf, Angeliz Rivera-Concepcion, Skylar McGaughey, Christina Yu, Dong Joon Lee, Stephen A. Tuin, Aya Ali

**Affiliations:** 1 Division of Comprehensive Oral Health - Periodontology, Adams School of Dentistry, University of North Carolina at Chapel Hill, CB# 7455, Rm 4610, Koury Oral Health Sciences, 385 S. Columbia St., Chapel Hill, NC 27599-7455 USA; 2 Oral and Craniofacial Health Sciences, Adams School of Dentistry, University of North Carolina at Chapel Hill, Chapel Hill, NC USA; 3 Adams School of Dentistry, University of North Carolina at Chapel Hill, Chapel Hill, NC USA

**Keywords:** Preclinical research, Physiology, Bone

## Abstract

Based on anti-inflammatory and osteogenic properties of hesperidin (HE), we hypothesized its systemic administration could be a cost-effective method of improving BMP-induced bone regeneration. Sprague–Dawley rats were allocated into 4 groups (n = 10/group): a 5-mm critical-sized mandible defect + collagen scaffold or, scaffold + 1 µg of BMP2 with and without dietary HE at 100 mg/kg. HE was administered by oral gavage 4 weeks prior to surgeries until euthanasia at day 7 or 14 post-surgery. The healing tissue within the defect collected at day 7 was subjected to gene expression analysis. Mandibles harvested at day 14 were subjected to microcomputed tomography and histology. HE + BMP2-treated rats had a statistically significant decrease in expression of inflammatory genes compared to BMP2 alone. The high-dose BMP2 alone caused cystic-like regeneration with incomplete defect closure. HE + BMP2 showed virtually complete bone fusion. Collagen fibril birefringence pattern (red color) under polarized light indicated high organization in BMP2-induced newly formed bone (NFB) in HE-supplemented group (*p* < 0.05). Clear changes in osteocyte lacunae as well as a statistically significant increase in osteoclasts were found around NFB in HE-treated rats. A significant increase in trabecular volume and thickness, and trabecular and cortical density was found in femurs of HE-supplemented rats (*p* < 0.05). Our findings show, for the first time, that dietary HE has a remarkable modulatory role in the function of locally delivered high-dose BMP2 in bone regeneration possibly via control of inflammation, osteogenesis, changes in osteocyte and osteoclast function and collagen maturation in regenerated and native bone. In conclusion, HE had a significant skeletal bone sparing effect and the ability to provide a more effective BMP-induced craniofacial regeneration.

## Introduction

Recombinant human bone morphogenetic protein 2 (BMP2) is a potent osteoinductive cytokine that plays a critical role in bone regeneration and has been studied extensively as a biologic for bone repair in dental and orthopedic fields^1–4^. The growth factor BMP2 is currently used as a bone-inducing agent for alveolar bone reconstruction, and several biomaterials and scaffolds have been introduced and evaluated as BMP2 carriers, e.g., absorbable collagen sponges, allogenic bone, bovine bone, hydroxyapatite and biphasic calcium phosphate^5^.

Although the usefulness of BMP2 for bone induction has not been questioned, the exponential rise in the clinical use of BMP2 (Infuse™ Bone Graft, Medtronic) use has been described to be associated with serious side effects^6^, such as inflammatory complications that may cause significant swelling and compromise a patient’s airway, cause ectopic bone, and tumor-like formation^7^. Other orthopedic-associated conditions like radiculitis, vertebral osteolysis, and increased microfracture occurrence has also been reported^8^. Therefore, it is important to find ways to better utilize BMP2 as a biologic either by controlling its inflammatory effects and/or potentiating its osteogenic function allowing for smaller quantities to be used^9–12^. Therapeutics that act in synergy with BMP2, limiting or avoiding side effects thus improving efficacy in many clinical applications, would be a paradigm shift in the field of regenerative dentistry and medicine.

Hesperidin (HE) is a natural compound found in citrus fruits that has been associated with various functions such as anti-inflammatory, anti-oxidative, anti-clastogenic and osteogenic^13–15^. Briefly, flavonoids present in citrus fruits, such as HE and its metabolites, have been shown to stimulate osteoblast differentiation through activation of BMP signaling^14,16–18^. Pre-clinical studies have demonstrated that HE intake results in bone density protection in senescent and ovariectomized rats as well as reduction in oxidative stress and overall lipid content^19–22^. In a human clinical trial, our group investigated the use of grape seed extract and grapefruit extract (the latter rich in HE) in bone healing of extraction sockets and found that grapefruit extract led to downregulation of inflammatory genes such as interleukin (IL)-1 β, IL-6 and CXCL2 in the healing tissue^23^. More recently, we reported the positive effects of this promising phenolic compound on pre-osteoblastic cell differentiation, on the quantity and quality of mineralization, and on the quality of the organic type I collagen-rich matrix in vitro*.* Such characteristics are critical for optimal bone properties as well as a bone-inducing role in vivo^12^. Our findings showed, for the first time, that HE has a modulatory role in mineralized tissue formation via promotion of osteoblast cell differentiation and improvement of matrix organization and mineral-to-matrix ratio. Moreover, the in vivo rat mandible regenerative bone model showed that HE combined with a suboptimal dose of BMP2 (a dose not able to promote fusion of the bone defect) in a collagenous scaffold promoted a well-controlled (not ectopic) pattern of bone formation as compared to a larger dose of BMP2^12^. Therefore, we have shown that HE delivered locally can promote BMP2 function suggesting that it could be a potential adjunct to the use of BMP2 in clinic.

Currently, there isn’t a way to effectively and consistently deliver HE concomitantly with BMP2 locally to a bone defect. The aqueous insoluble nature of hesperidin without a delivery system to address solubility may pose standardization of treatment difficult at this time. We proposed to evaluate the effect of systemic administration of HE as a dietary supplement to BMP2/collagen scaffold-induced (locally delivered) bone regeneration to circumventing these limitations. The use of HE for local delivery warrants further investigation of a release system such as nanoparticles, branched polymers, or hydrogels with predictable rates of degradation to facilitate its solubility and ensure cell and matrix uptake as well as concomitant BMP2 delivery^12^. Since HE is already available as an over-the-counter (OTC) supplement, dietary administration of HE could, in the meantime, be a practical and immediate alternative to modulation of BMP2-based regeneration while local delivery system(s) are being developed. OTC supplementation could be utilized as adjunctive and concomitant treatment during bone graft procedures in dentistry and orthopedics.

Thus, taking into consideration the role of HE in inflammation and in osteogenesiss/osteoclastogenesis, our study aimed to evaluate bone healing in a critical-sized mandible defect of rats under HE supplementation by evaluating early inflammatory/osteogenic gene expression and histopathological and tomographic bone parameters in bone healing sites treated with or without BMP2. Our hypothesis was that systemic HE supplementation would positively modulate inflammation associated with use of high-dose BMP2, promote osteogenesis, influence osteoclastogenesis and favor mandible bone quality, ultimately leading to a more desirable regenerative therapy free of abnormal and poor-quality bone formation. Since the rats would be on dietary HE for 6 weeks, off target effect on skeletal bones were anticipated, thus, femurs were imaged for effect on skeletal bone mass. This report is the first in the literature showing a benefit in consumption of the dietary compound HE during BMP2-induced bone healing.

## Materials and methods

### Animals and groups

The Institutional Animal Care and Use Committee (IACUC) at the University of North Carolina at Chapel Hill (IACUC ID: 18–115, 04/30/2021) approved the animal experiment protocol and the procedures were conducted in compliance with ethical standards that fully comply with Animal Research: Reporting of in vivo Experiments (ARRIVE) guidelines^24^.

Forty male Sprague–Dawley rats (350–400 g, 70 d old) were randomly allocated into 4 groups (n = 10/group): mandible defect + scaffold with (1) or without (2) HE 100 mg/kg dietary supplementation and, mandible defect + scaffold with 1 µg of BMP2 with (3) or without (4) supplementation with HE 100 mg/kg. The animals were kept in a BSL 2 classified animal environment with controlled temperature, humidity, and light cycles (12/12 h), with water and feed ad libitum. The diet consisted of hard chow provided by the facility at all times, with soft chow (hard chow soaked in water) for 48 h post-surgery.

### Hesperidin treatment

HE (97% purity, ACROS Organics, Belgium) concentration at 100 mg/kg of rat weight was diluted in 0.9% sodium chloride mixed fresh at time of ingestion and administered systemically once daily via oral gavage by experienced personnel as reported^25^. HE treatment started 4 weeks before mandible surgery and was maintained until euthanasia at day 7 or 14 post-surgery. The non-HE rats did not receive sham gavage.

### Mandible model and femur phenotype

**Figure 1:**
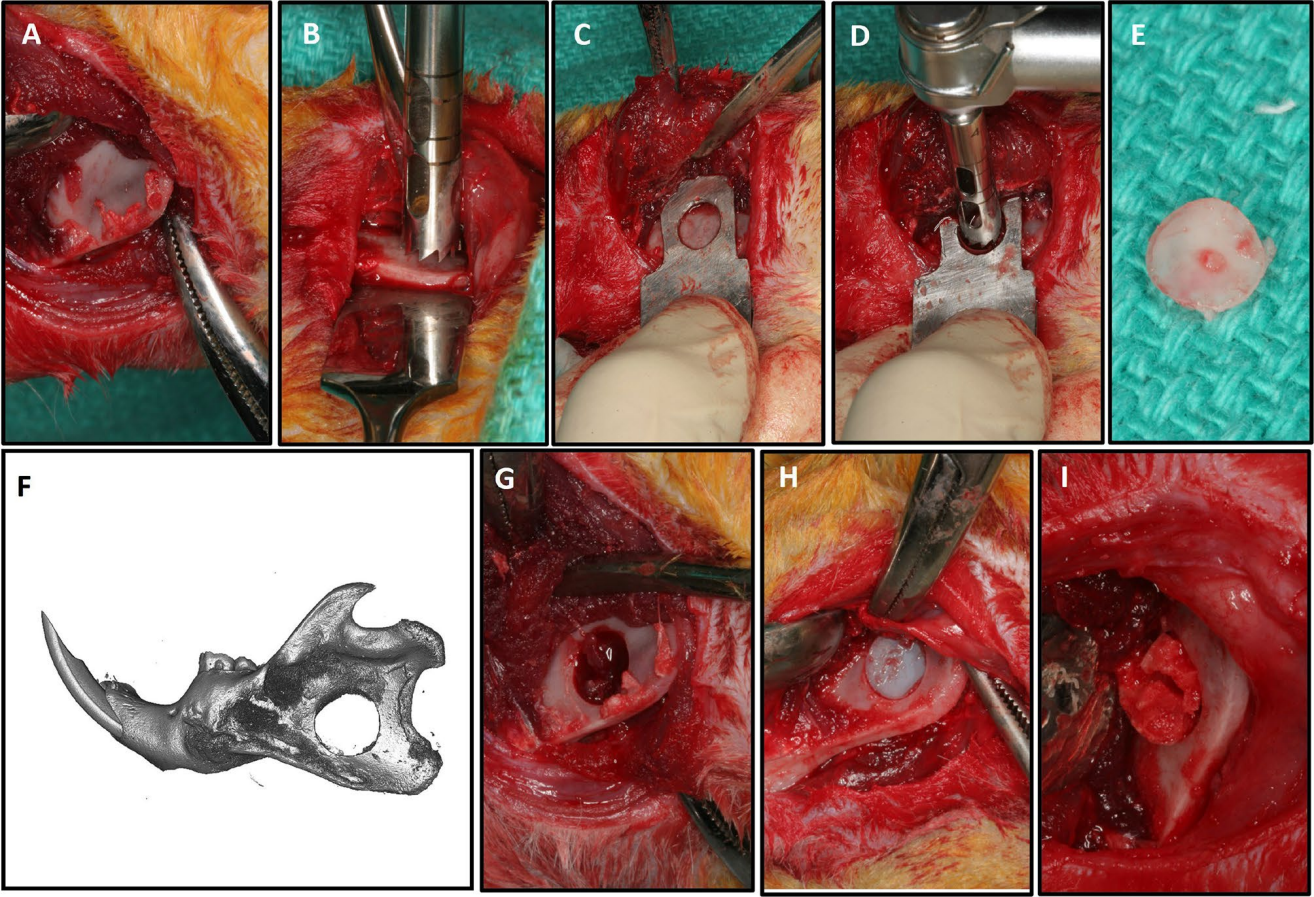
Critical-sized defect of the rat mandible. (**A**) Surgical exposure of the lower body, angle and ramus of the mandible after dissection of masseter and medial pterygoid. (**B**) 5-mm trephine alone can be used to generate the 5-mm critical-sized defect using a Prichard instrument over the sagittal side of the mandible for protection of the underlining soft tissue. (**C**) Alternatively, a custom-made template (prototype is shown) is recommended to standardize the location of the defect and protect underlying tissues (jig archetype available at otc.unc.edu, Tech#20–0105). (**D**) The trephine fits within the open window of the jig. (**E**) The bone removed has well-defined edges and is from the same location in all mandibles. (**F**, **G**) Critical-sized defect. (**H**) Placement of a 5-mm collagen scaffold in the defect. (**I**) The scaffold can be removed within a week post-surgery for analysis of bone healing such as via mRNA extraction and gene expression profile mapping.

As previously described (Miguez et al.^9,10,12^), all animals were given a pre-operative dose of the antibiotic Cefazolin (10 mg/kg), and the anesthesia was achieved by using Ketamine (80 mg/kg)/Xylazine (10 mg/kg). Two-cm incisions were made along the inferior border of the hemi-mandibles and the masseter muscle, and the periosteum, were detached to expose the ramus (Fig. 1A). Using a 5-mm-diameter trephine (Salvin Dental, Charlotte, NC, USA), through a mandible jig prototype (archetype jig: http://otc.unc.edu, Tech#20–0105) a critical-sized defect was placed at the ramus at a jig-oriented standardized location about 3 mm above the lower border of the mandible and 2 mm distal to the incisor root (Fig. 1B–D). A 5 mm bone core was removed without rigged borders and a well-defined critical-sized defect was obtained at a standardized and reproducible location (Fig. 1E–G). The defects were filled with a UV-cross-linked type I collagen sponge (Nitta Gelatin, Japan) as a scaffold. Each scaffold was precut with a 5-mm-diameter tissue punch (Miltex Inc., York, PA, USA) and was soaked uniformly in 10 µl total solution of phosphate-buffered saline (PBS) with and without the growth factor BMP2 (rhBMP2, catalog #:355-BM, R&D Systems, Minneapolis, MN). Two groups received an empty scaffold pressed into the defect, while the other two groups, the bone defect was filled with the scaffold soaked with 10 µl PBS containing BMP2 at 1 μg concentration (Fig. 1H). The muscle layer was passively, but tightly, sutured around the mandible with 5–0 chromic gut (Ethicon Inc., Cornelia, GA, USA) and the skin with 5–0 polypropylene suture (Ethicon). The rats were maintained on a diet of soft rat chow and water for 48 h and starting on day 2 had both soft and hard diet available. Rats received buprenorphine for pain management. Any complications such as seromas, signs of distress or change in behavior were recorded and managed immediately to avoid animal stress and discomfort.

At day 7 post-surgery, animals (n = 5/group) were anesthetized by ketamine/xylazine, the surgical access to the mandible was re-opened and the collagenous tissue present within the mandible bone defect was collected and immediately stored in an RNA stabilization solution (RNA *later®,* Sigma-Aldrige, Burlignton, MA) (Fig. 1I) until processed for gene arrays. Another cohort of animals (5/group) was euthanized at day 14 post-surgery and the mandibles were immediately fixed in 10% neutral buffered formalin for 72 h, rinsed in PBS and stored in 70% v/v ethanol at 4 °C. Femurs of the groups treated with or without HE were also harvested 2 weeks post-surgery, to evaluate the impact of 6 weeks of systemic dietary HE on femoral phenotype. All animals were sacrificed according to the IACUC and ARRIVE guidelines with a lethal dose of pentobarbital followed by the physical method of thoracotomy. The formalin-fixed mandibles with intact attached muscle and femurs were analyzed by microcomputed tomography (µCT). Mandibles were demineralized with 0.5 M EDTA at pH 7.4 for 8 weeks under agitation, paraffin-embedded and processed for histology.

### RNA isolation and PCR arrays

Seven-day samples stored in RNA later were processed for RNA extraction via Trizol (Invitrogen, Carlsbad, CA) as described previously (Miguez et al.^12^). RNA was further purified using the miRNeasy Mini Kit (Qiagen, Germantown, MD) and RNA integrity assessed using a NanoDrop ND-1000 spectrophotometer (NanoDrop Technologies, Wilmington, DE). The cDNA synthesis and gDNA elimination were performed using the RT^2^ Microfluidics qPCR Reagent System (Qiagen) with 100 ng (first experiment)/500 ng (second experiment) RNA used as input. Next, specific target preamplification was performed using the RT^2^ PreAMP Primer Mix Format H (Qiagen) for the Rat Osteogenesis (GeneGlobe ID—PBR-026Z) and Rat Inflammatory Cytokines and Receptors (GeneGlobe ID-PBR-011Z) Arrays. Finally, real-time PCR was performed using the RT^2^ Profiler PCR Array Format H (Qiagen), Rat Osteogenesis and Rat Inflammatory Cytokines and Receptors Arrays, and Microfluidics qPCR Master Mix (Qiagen) on the BioMark HD system and PCR Analysis Software v2.1 (Fluidigm). The log base two fold-change ratios were calculated and clustered data defaulted using blue and red to represent positive and negative fold-change expression values, respectively.

All steps were performed according to manufacturer's instructions as reported^26,27^.

### Microcomputed tomography bone analysis

The mandibles were scanned using a µCT system (Scanco µCT40 scanner—SCANCO Medical AG, Bruttisellen, Switzerland)^12^. The X-ray parameters were 70kVp at 114uA with a 200 ms integration time. Image matrix size was 2048 × 2048 with acquired 2000 projections over a 360° rotation. A tube of 20.5 mm diameter which allows a pixel size of 10 μm was used. Acquisitions were made using a conebeam geometry and a Feldkamp filtered backprojection reconstruction algorithm used to create the reconstructions. All samples were positioned and scanned in a standard manner using an airtight cylindrical sample holder filled with PBS. For the analyses of the acquired images, the CTAn analyser software (Skyscan) was used. The region of interest (ROI) was selected in all images through a standardized drawing of the area within the defect borders. The region was first positioned in the middle of the surgical defect, and it was then extended to all slices of the data set. A new ROI was set every 20 images. Quantitative morphometric analysis of the mineralized tissue inside the defects was carried out on voxels that corresponded to bone. After tomographic acquisitions, 3D images were reconstructed through direct volume rendering from the series of 2D projections^9,10,12^. For the femurs, a 6 µm, 70 kVp, 142 uA using a 0.5 mm aluminum attenuation filter setting was used. The ROI included a specific number of slices below the growth plate after a set anatomical marker for trabecular bone and cortical bone analysis (50 and 400 slices below growth plate, respectively). The bone microarchitecture parameters evaluated were trabecular and cortical bone volume fraction (BV/TV, %), trabecular thickness (Tb.Th), trabecular number (Tb.N), trabecular separation (Tb.S), and trabecular and cortical density (mgHA/ccm) as previously recommended^28^. A threshold of 330–1000 was used to separate mineralized and non-mineralized tissue in both mandible and femurs with cortical bone with a minimal threshold of 360.

### Histological analysis

After mandibles were demineralized and embedded, sections (6 µm) obtained from the mid–cross section of the mandible in the frontal plane were stained by Hematoxylin and Eosin (H&E) for a qualitative gross evaluation and picrosirius red (PSR) for quantitative collagen organization and maturation analysis. Osteoclast numbers were assessed by tartrate-resistant acid phosphatase (TRAP) staining as previously described^10,25^.

For PSR, slides were incubated in 0.1% (w/v) sirius red in saturated picric acid solution for 30 min at room temperature. This was followed by rinsing with distilled water, dehydration and mounting. The slides were imaged under bright field and polarizing light using a Leica DMR microscope (Buffalo Grove, IL, USA). PSR images at 20 × magnification were analyzed using a custom generated algorithm in Matlab ® R2016a (Mathworks, Natick, MA, USA) as previously reported (Smith et al.^29^, Miguez et al.^12^). The percent area of red, yellow, and green color signals were normalized to total color signal for each sample.

Non-serial histological sections (n = 5/animal) were stained for TRAP-positive multinucleated osteoclasts, and slides were deparaffinized and rehydrated through graded ethanol to distilled water, and then immersed in TRAP staining solution mix (at 37 °C for 30 min). The sections were rinsed in distilled water and counterstained with 0.02% fast green for 30 s. For quantification, TRAP-positive cells stained in red containing three or more nuclei on the bone surface, were considered osteoclasts, and quantified in the region of interest comprising the whole area within the defect borders^25^.

### Statistical analyses

An evaluator from a bioinformatics core performing the statistical analyses was blinded to the identity of experimental groups for gene arrays. Gene arrays were evaluated by Analysis of variance (ANOVA) and Tukey post hoc as described^27^. Data were submitted to the Shapiro–Wilk test to assess homogeneity and data distribution. ANOVA with Tukey post hoc test or Kruskal–Wallis with Dunn post hoc test were performed to determine the differences among groups of animals for µCT and osteoclast numbers as described^10,12^. PSR colors were quantified by MatLab software as described^12,29^ and statistically evaluated by ANOVA with Tukey post test. GraphPad Prism 8 software (GraphPad Software Inc., San Diego, CA, USA) was utilized for analyses. All tests were applied with a 95% confidence level (*p* < 0.05). Data were expressed as mean ± standard deviation.

## Results

### Adverse events

Systemic HE administration, surgical procedures and scaffolds did not promote any behavioral change, physical issues (e.g., skin lesions, hair loss), detrimental weight loss or feeding impairment**.** Three animals in the BMP2-treated group developed seromas in the submandibular space which were monitored as the animals were not stressed and were ambulating, behaving, and eating normally.

### Hesperidin influence in inflammatory and osteogenic genes

**Figure 2:**
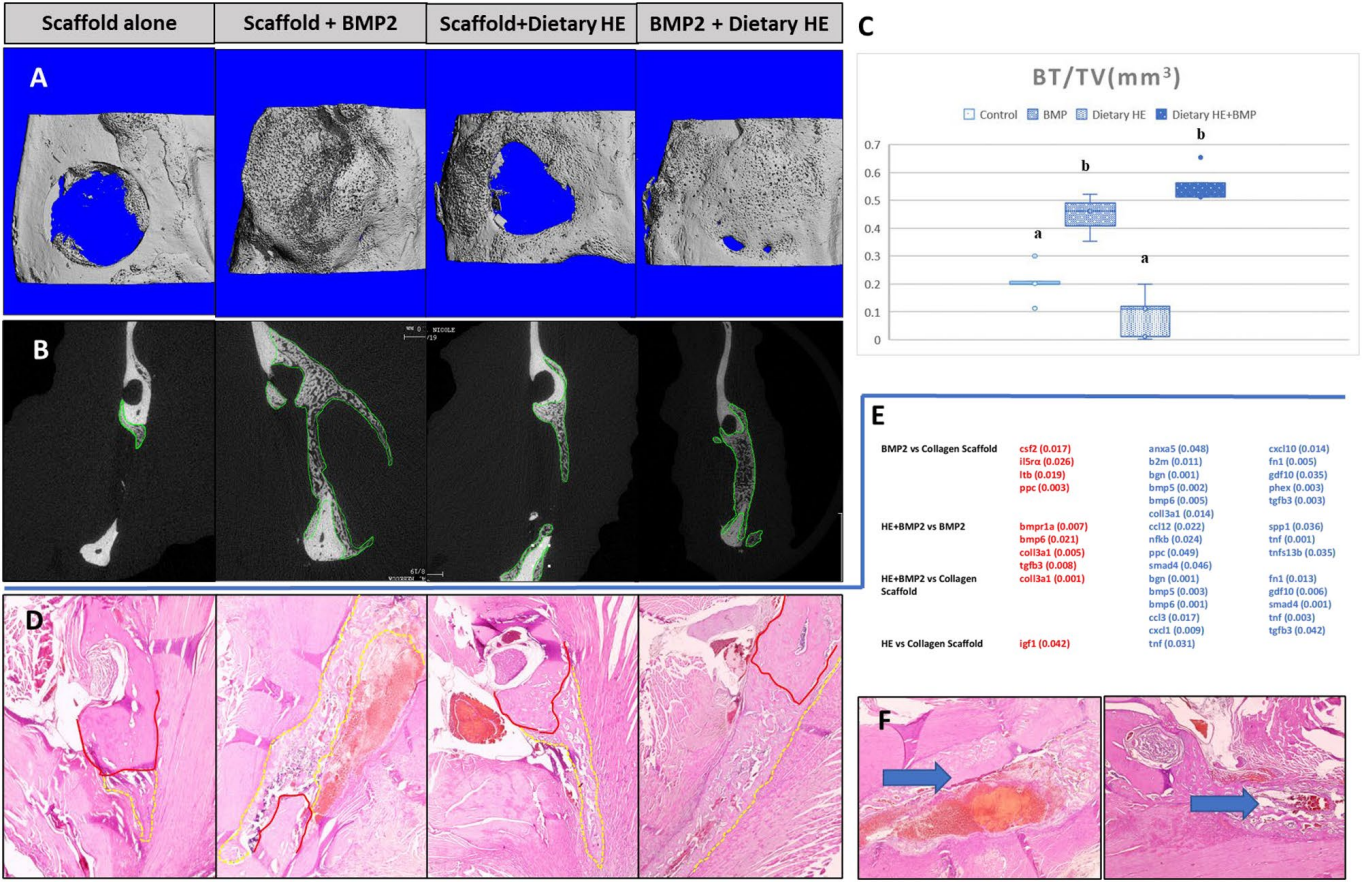
Assessment of bone healing and regeneration of the rat mandible via inflammatory genes arrays, microcomputed tomography (µCT) and histology when using bone morphogenetic protein 2 (BMP2) with and w/o dietary hesperidin (HE). (**A**) 3D images show increased bone formation with use of BMP2 (dose previously found to induce enough bone regeneration to fuse the defect in this model-1 µg of BMP2, albeit with cyst-like bone formation) compared to collagen scaffold alone. When HE was administered systemically in rats via oral gavage, BMP2 formed bone within the boundaries of the defect only vs. of ectopic or cystic nature with BMP2 w/o HE supplementation. (**B**) 2D view of coronal cross-section of the mandibles confirmed the 3D image bone profile to be abnormal when BMP2 was used w/o dietary HE as opposed to with HE. HE formed a thick and trabeculate type of bone within the constraints of the defect. (**C**) There was no statistical difference in BV/TV between defects treated with BMP2 with and w/o HE but both groups are statistically superior in BV (n = 5, *p* < 0.05, different superscript letters indicate statistical difference among groups) to control scaffold. (**D**) Histological sections stained with H&E with native mandible bone delineated in red and newly formed bone in dotted yellow. (**E**) Gene profile of samples collected from BMP2, HE + BMP2 and control scaffold alone (n = 4 scaffold, n = 5 −BMP2, n = 3 scaffold + HE, n = 4 HE + BMP2); blue lettering indicates downregulation, red lettering indicates upregulation, all samples ran in triplicates. (**F**) Note that the inflammatory infiltrate and blood-filled cavity within bone is histologically evident in BMP2 alone samples (blue arrow). One-way analysis of variance, Tukey’s post hoc test performed for imaging and gene expression parametric data at 95% confidence interval.

Figure 2 depicts the statistically significant up and downregulated osteogenic and inflammatory gene findings and their p values. BMP2 in high dose in the rat mandible model compared to scaffold alone after 7 days of healing, significantly decreased the expression of several genes including biglycan (*bgn)*, *bmp6*, *bmp5*, fibronectin (*fn1*), the main BMP2 receptor gene *bmpr1a*, c*oll3a1*, *anexin5* and *phex* . One of the most significant downregulation in BMP2 treated rats vs. scaffold alone was *tgfb3*. BMP2 increased inflammation-associated genes such as *ltb* and *il5rα*.

When rats were on dietary HE and subjected to BMP2-induced bone regeneration (HE + BMP2) were compared to BMP2, the healing tissue showed a statistically significant decrease in expression of the inflammatory genes *tnf*, *nf-*κ*b* and *ccl12*. Interestingly, expression of *smad4* and *spp1* (osteopontin) were significantly reduced potentially indicating some control of osteogenesis via HE. *Coll3a1* was significantly upregulated as well as *tgfb3*, *bmpr1a* and *bmp6* in HE-rats.

Compared to scaffold alone, the HE + BMP2 mostly downregulated gene was the BMP function promoter *bgn*, followed by *tgfb, bmp6*, *bmp5*, *tnf*, *gdf10*, *cxcl1*, *smad4*, *fn1* and *ccl3*. Only *coll3a1* expression was statistically significantly increased. Scaffold with and without dietary HE showed statistically significant increased expression of *igf1* and decreased expression of *tnf* (*p* < 0.05).

### HE administered systemically promotes a more robust and well-defined BMP2-induced bone regeneration

Three and two-dimensional analysis (Fig. 2A–C) of newly formed bone within the defects showed a small percentage of bone formation in samples treated with scaffold only in both groups with and without HE supplementation. A significantly robust and cyst-like bone formation was observed in the defects treated with scaffolds infused with 1 µg of BMP2 compared to no growth factor. Bone volume was also significantly increased compared to scaffold alone in rats under dietary HE regimen and treated with BMP2 (*p* < 0.05) but was not different compared to BMP2 scaffold without HE. Remarkably, the bone formation in the HE-rats virtually closed the mandible defect without any evidence of abnormal bone formation such as cysts or ectopic sites in all rats. Histological evaluation (Fig. 2D) by H&E confirmed the pattern of bone regeneration seen on µCT and revealed pronounced inflammatory infiltrate associated with BMP2 groups without HE treatment including heme-filled cavities (Fig. 2F)**.**

### Dietary HE supplementation leads to a more mature extracellular matrix

**Figure 3:**
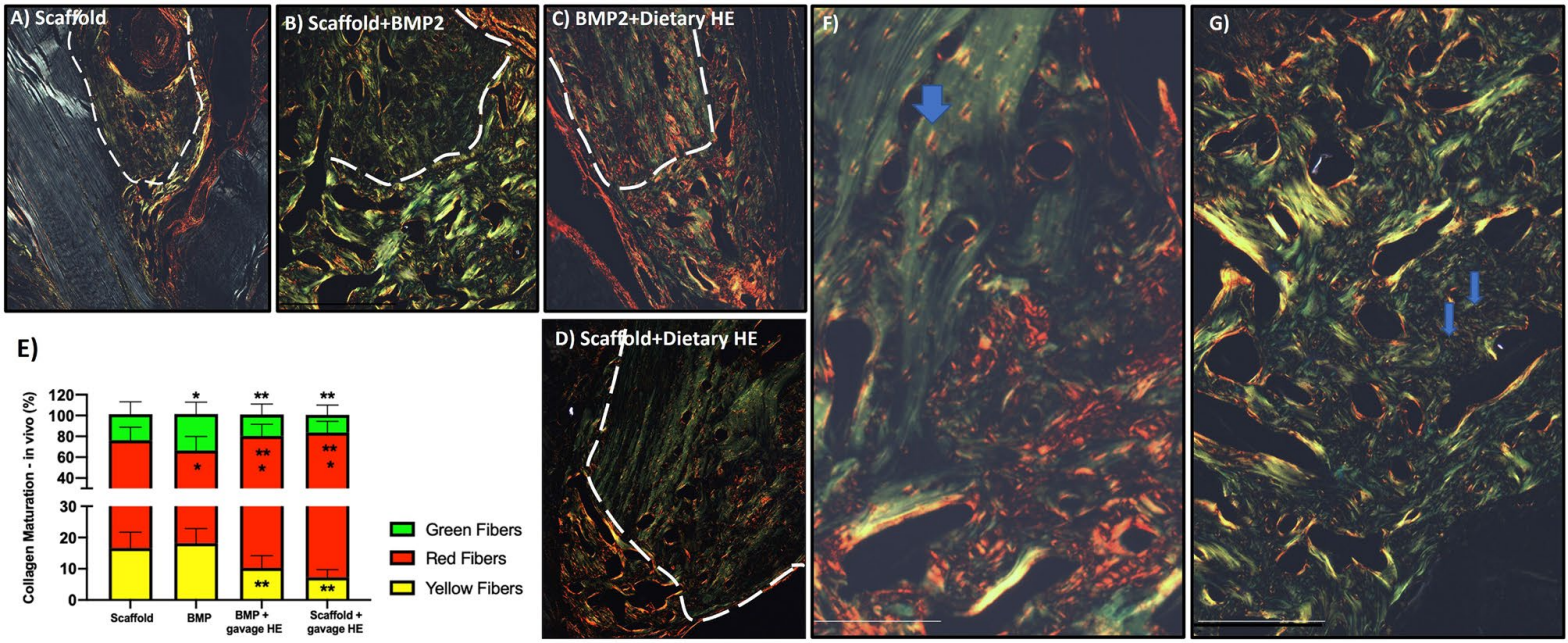
Assessment of extracellular matrix organization via picrosirius red (PSR) staining of rat mandibles. Native bone at the edge of the mandible appears within white dotted line. (**A**) Upper border of the mandible defect treated with scaffold alone shows small area of bone formation in faint green/yellow birefringence pattern at 40× magnification. (**B**) Extensive green staining pattern of newly regenerated bone when 1µ BMP2 was infused in scaffold (no HE supplementation). (**C**) In the presence of dietary HE supplementation, BMP2-induced regenerated bone showed evidence of red-colored collagen fibrils. (**D**) NFB in all HE-treated animals showed statistically greater red color appearance. Differences statistically significant as quantified by MatLab software and analyzed by ANOVA and Tukey post hoc test (n = 8, *p* < 0.05) are depicted in (**E**) *p < 0.05 compared to scaffold group; **p < 0.05 compared to BMP group. (**F**) At 200x, note that in HE-treated rats, the polarized imaged area around osteocytes (lacunae) is marked by yellow and red matrix suggesting active osteocyte function even in the native bone area (blue arrow) as opposed to the much less-defined osteocyte lacunae of rats not on dietary HE (**G**). Scale bar indicate 200 µm.

PSR staining showed a quantifiable pattern of mature collagen fibrils of predominantly red birefringence with a lower percentage of green and yellow fibrils in BMP2-treated rats while supplemented with HE as compared to only scaffold or BMP2 alone. Dietary HE promoted red colored fibrils compared to non-HE consuming animals (*p* < 0.05) (Fig. 3A–D,E).

Osteocytes lacunae were clearly dissimilar in rats under HE supplementation compared to no HE. The lacunae was marked by intensive sirus red staining (dense and of mostly unidirectional collagen matrix) revealing a spindle shape form of the osteocyte cavity compared to less defined, poorly collagen-dense walls and round-shaped lacunae for non-HE rats (blue arrows, Fig. 3F,G).

### Osteoclasts numbers are increased in NFB of HE-treated rats at 100 mg/kg

**Figure 4:**
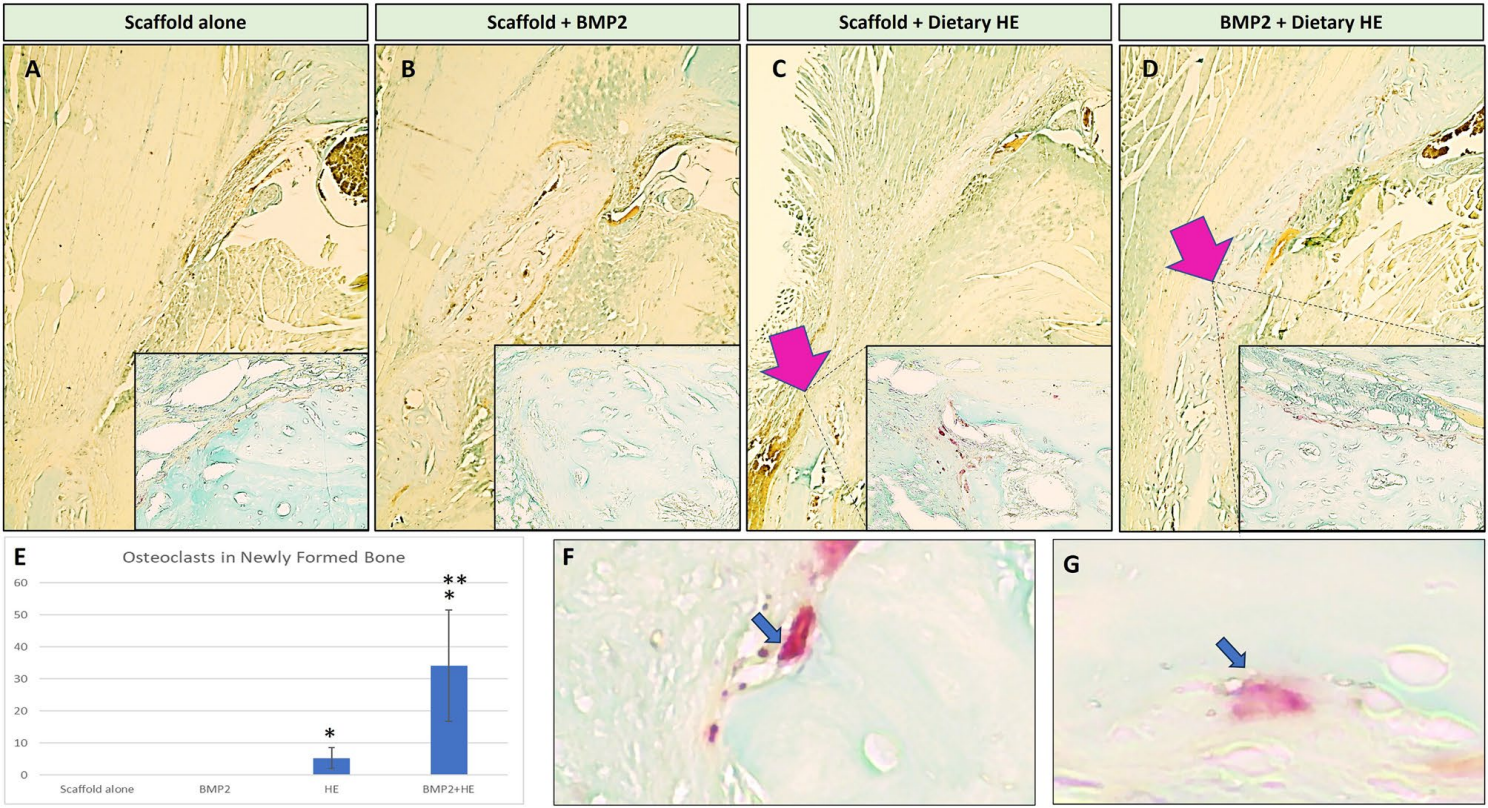
TRAP staining for osteoclast identification in newly formed bone of the rat mandible defects 2 weeks post-surgery. (**A**) Scaffold alone (large figure, 4×; insert 40×). (**B**) Scaffold + BMP2 showed no quantifiable osteoclast staining. (**C**) Use of dietary hesperidin (HE) was associated with presence of several osteoclasts lining the regenerated bone (pink arrows). (**D**) Dietary HE in combination with BMP2 showed a larger area of active osteoclasts surrounding the newly formed bone. (**E**) Quantification of osteoclasts by TRAP staining showing statistically significant differences among scaffold + HE and HE + BMP2 groups compared to scaffold alone (*) and among BMP2 and BMP2 + Dietary HE groups (**) (n = 5–7 slides per animal, 4 animals, *p* < 0.05). (**F**) and (**G**) are representative images of TRAP + cells with 3 nuclei or more, which were accounted for as osteoclasts.

TRAP staining (Fig. 4A–D) showed that there were no osteoclasts present at 2-weeks post-surgery in the NFB area indicating no detectable active remodeling in BMP2-treated or scaffold alone groups. Interestingly, only in the presence of dietary HE, it was possible to find osteoclasts in the samples localized to the surrounding area of the NFB 14 days post-surgery (Fig. 4C,D). BMP2 + HE + BMP2 rats showed the highest number of osteoclasts (34.1 ± 17.4) compared to HE (5.3 ± 3.1) which were statistically significant from non-HE samples (*p* < 0.05, n = 5–7 slices/rat, n = 4 rats, Fig. 4E). TRAP positive and multinucleated cells were identified as osteoclasts (Fig. 4F,G).

### Trabecular and cortical femoral bone parameters are improved by dietary hesperidin

**Figure 5:**
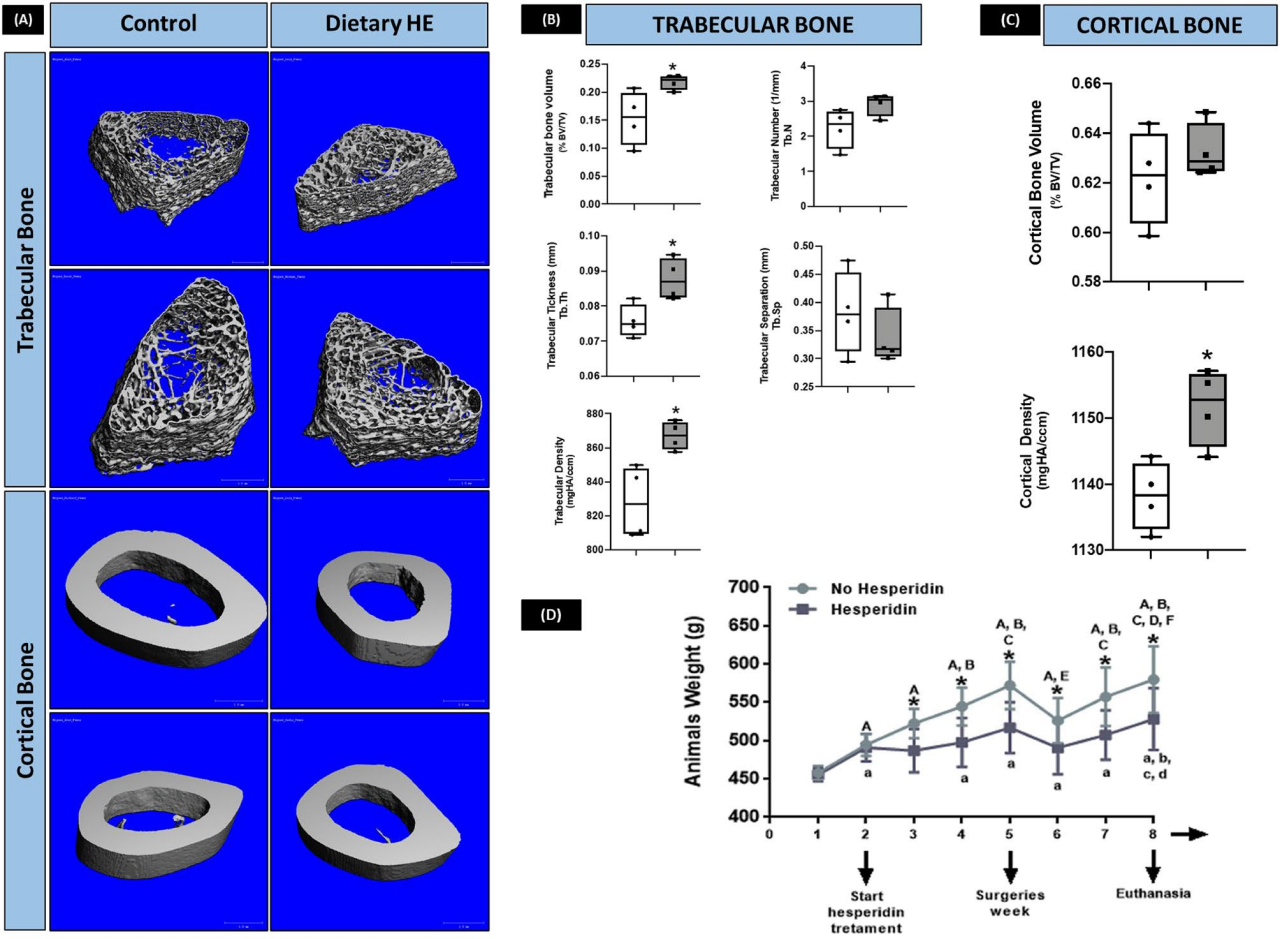
µCT evaluation of femoral bone and animal weight after 6 weeks of dietary hesperidin (HE) supplementation. (**A**) Representative images per group of 3D reconstructed models of femurs, and microarchitecture parameters of trabecular (**B**) and cortical bone (**C**). (**D**) There was a significant reduction in weight of the HE-treated animals during the course of 6 weeks. µCT analysis showed HE promoted a bone-sparing effect on femoral bones as shown by the trabecular and cortical bone parameters. *p < 0.05 comparing the same week between no HE (white box-plot) and HE (gray box-plot). Weight analysis where upper case letters indicate *p* < 0.05 comparing different weeks in control mice (no HE). Lower case letters indicated statistical difference between different weeks in HE animals as compared to week 1.

Systemic administration of HE significantly affected femoral bone parameters 6 weeks-post introduction of the oral gavage with HE (Fig. 5A–C). Trabecular bone volume (BV), trabecular thickness (Tb.Th.) and mineral density were significantly increased by HE when compared to no HE (*p* < 0.05). HE administration also promoted a slight but significant increase in cortical bone mineral density compared to control group (no HE treatment).

Of note, rats under HE-regimen lost significant amount of weight after starting the oral gavage (although no measurable difference in food intake was noticed in the first week, Fig. 5D). In the subsequent weeks, HE-rats maintained the statistically significant lower weight compared to no HE, were alert and had normal behavior and eating patterns. Both groups lost weight the week after surgery as expected due to the nature of the surgery with limited food intake in the first days post intervention.

## Discussion

The incorporation of BMP2 has been found to remarkably enhance the bone restorative effect of synthetic bone substitutes and greatly expand the horizon for the clinical application of bone grafts^30^. However, BMP2 tends to be rapidly degraded by proteases when injected directly into a defect site and, thus, a supraphysiological dose of the protein is typically required. This dose can cause undesired side effects^31^, e.g., excessive inflammation, occasional ectopic ossification, tumor-like bone growth, and edema^12,32–34^.

As previously demonstrated in our studies, when delivered locally to a critical-sized defect in a rat mandible model, HE combined with a suboptimal dose (not enough to regenerate the defect) of BMP2 in a collagenous scaffold, promoted a well-controlled pattern of bone formation as compared to a large dose of BMP2^12^. Complementary, the present study is the second evidence showing HE effects on BMP2-induced regeneration and the first evaluating dietary HE on BMP2-bone therapy. Defects filled with a high dose of BMP2 in a collagenous scaffold only formed bone within the boundaries of the mandible defect when HE was administered systemically over the course of the healing period, which is in agreement with the results observed when HE was associated to BMP2 and directly delivered into bone defects^12^. HE effects observed in both models are paradigm changing and very promising in the pursuit of cost-effective regenerative medicine and dentistry. The remarkable effects of HE in controlling BMP function could be associated with its modulation of osteogenic, resorptive and inflammatory cell functions^12,17,22,35^.

Indeed, an inflammatory profile has been observed with BMP2 use in the rats in this and previous studies histologically. The literature has described an exaggerated inflammatory response after implantation of BMP2 in an absorbable collagen sponge in animal models or clinical settings^36–41^. A remarkable reduction in *ccl12, nfkb* and *tfn* pro-inflammatory genes were found where HE was present systemically in rats treated with BMP2 compared to no HE. Corroborating our result, HE was described to attenuate alteration in lung NF-κB expression in rats^42^, while the topic application of 5% HE hydrogel decreased NF-κB expression in granulation tissue of skin wounds in mice^43^. In a mouse model of gout arthritis, HE inhibited NF-κB activation^44^. NF-κB signaling pathway is one of the most well-studied pathways related to inflammation, since this transcription factor regulates the expression of various genes involved in inflammation and the immune response (including TNF and CXCL2)^45–47^. The importance of NF-κB in both bone formation and bone resorption is well-known^48^, and genetic mutations in molecules involved in the NF-κB signaling pathway in mammals cause pathological bone phenotypes, including osteopetrosis^49^. Of the inflammatory genes, *ccl3, cxcl1* and *tnf* genes, were also significantly downregulated in HE + BMP2 rats compared to scaffold alone. Previous evidence has reported a regulatory effect by different classes of flavonoids (apigenin, quercetin, fisetin, astragalin, HE, hesperitin) in the chemokine subfamilies CXC and CC, and TNF activity^50–59^. Comparison of the scaffold groups with and without dietary HE, showed that *tnf* had statistically significant reduced expression as well. Our results share similarities with prior evidence demonstrating an anti-inflammatory effect of HE during bone formation in our mandible model. In contrast, evaluating the systemic effects of HE in a model of periodontal inflammation^25^, this flavonoid increased the inflammatory profile, tissue damage and bone resorption in rats with periodontal disease, which can in part be attributed to the characteristics of the model studied. As previously described by de Paiva Gonçalves et al.^25^, the complexity of the diseased environment, local inflammation, as well as flavonoid dosage, are crucial factors to be considered in the cell responses to HE. Nonetheless, the remarkable reduction in the inflammatory cell influx, the evidence of organized, high quality and non-ectopic healing bone in rats treated with HE at 100 mg/kg + BMP2 at 1 µg are likely the direct result of a primarily non-hyperinflammatory healing site.

Our previous studies have demonstrated an osteogenic function of HE when used in combination with a low dose of BMP2 (both delivered locally)^12^. In this study, the use of systemically administered HE with high-dose BMP2 compared to rats not on HE and treated with the same dose of the growth factor, led to a substantial and significant decrease in *osteopontin* and *smad4* expression, important in osteogenic signaling and mineralization. There was also an increase in *coll3a1* (important in collagen maturation and angiogenesis), *tgfb3* (known to control BMP function and matrix deposition and can control collagen crosslinking), *bmpr1a* (a known receptor for BMP2 signaling), and *bmp6* (important in endochondral ossification)^60–67^.

The significant reduction observed in genes such as *smad4* and *spp1* (as well as *bgn* and *bmp6* compared to scaffold alone) gene expression by HE-treated animals, is curious, as those are important in osteogenesis. Smad proteins mediate the signal transduction in TGF and BMP signaling pathways, affecting both osteoblast and osteoclast function, and therefore play a critical role in the regulation of bone remodeling^68^. BGN is detected at high levels in areas of endochondral and intramembranous bone formation^69^ and has been extensively investigated in its role in promoting BMP function, vascularization and more recently, inflammation^9,69–71^. A previous study^72^ showed that flavopiridol, another flavonoid, inhibits TGFβ-stimulated BGN synthesis by blocking linker region phosphorylation and nuclear translocation of smad2 in vascular smooth muscle cells. There seems to be a fine-tuned HE-mediated control of bone formation which is involving many key osteogenic players such as TGFβ3 and, in turn, *bmpr1α*, *smad4, spp1* and *bgn* gene expression^61^.

It is important to highlight how the high-dose BMP2 affected the healing bone compared to the collagen scaffold alone. It mostly decreased the expression of *bgn* (a BMP2 and 4 function promoter), *bmp5* and *bmp6* (which are associated with induction of chondrogenesis), *fn1* (of key importance to cell adhesion, migration, growth and differentiation), *bmpr1a* (likely controlling osteogenic function due to over activation of BMP receptors), *coll3a1* (involved in collagen organization and angiogenesis), and *phex*^60,65,67,70,73,74^. High dose BMP2 also significantly increased *ltb* (a chemoattractant of leukocytes), and decreased (with function largely unknown but thought to modulate coagulation) and *cxcl10* (suggested to promote osteoclast differentiation)^75^. Both *ltb* and *anexin5* could correlate with the high presence of heme in the cystic area and hematomas associated with some rats^76,77^.

PSR showed that collagen fibrils birefringence was mostly red for HE-treated animals. Typical staining of collagen via PSR shows red, yellow, and green colors which have been associated with collagen packing and post-translational modifications. Red indicates more mature/well-packed/organized collagen and green least mature/loose-packed/disorganized^29,78^. This assessment is important as collagen post-translational modifications have been associated with proper tuning of mineralization and mechanical properties of tissues such as bone^79^. In addition, PSR staining showed very defined, active areas around osteocytes in the presence of systemic HE. However, without HE, BMP2-treated bone had non-distinct lacunae (characterized by poor collagen packing and mineralization). BMP samples showed a significant decrease in *phex* gene expression. Phex is important in cleavage of proteins such as osteopontin, is expressed in early and mature osteocytes and is involved in phosphate metabolism as well as growth of bone. Decrease in phex may mean poor phosphate clearance, altered mineralization and may be important in controlling remodeling^80^.

The clear increase in *coll3a1* expression by systemic HE treatment, could be correlated and potentially influenced by active osteocytes as well, considering that those cells synthesize several proteins involved in bone formation such as Type I collagen, osteocalcin, sclerostin and Dkk-1^81^. Through this mechanism, the suggested active osteocyte function may contribute to a well-defined and mature bone healing pattern. Osteopontin (OPN) is a highly phosphorylated and glycosylated sialoprotein that is expressed by several cell types including osteoblasts, osteocytes, and odontoblasts^82^. This non-collagenous protein is encoded by the *spp1* gene and is involved in biomineralization of bone tissue, bone remodeling, wound healing and apoptosis^82,83^. OPN was described to contribute to bone remodeling by promoting osteoclastogenesis and osteoclast activity through CD44- and αvβ3**-**mediated cell signaling. Further, it plays a definitive role in bone remodeling by the formation of podosomes, osteoclast survival, and osteoclast motility^82^. Mirroring these considerations, the significant decrease of *spp1* gene expression encoding OPN by HE, may constitute a mechanistic pathway to control remodeling (and phex increase also would decrease *spp1* availability in BMP2 alone). Furthermore, the polarized imaged area around osteocytes (lacunae) in HE-treated groups, is marked by yellow and red matrix, suggesting active osteocyte function even in the native bone area. OPN is highly observed in the osteocytes during initial stages of orthodontic tooth movement^84^, and a model of mechanical stress shows that the number of OPN-mRNA expressing osteocytes on the pressure side after 48 h of mechanical stress reach a maximum value at 72 h, coinciding with bone resorption^85^. Therefore, the increased osteocyte activity could also be contributing to the marked increase in osteoclast presence in HE-treated rats.

Correlated with the mandibular bone regeneration results, HE presented significant effects on femoral bone parameters 6 weeks-post flavonoid intake. HE improved trabecular and cortical bone microarchitecture, as previously observed in a similar study in rats performed by our group^25^. However, the femur model was limited to assessment of the tomographic parameters, thus we are not able to conclude if the effects of HE at 6 weeks were due to effect on rate of osteoclastogenesis and/or osteogenesis in long bones. In future studies, the kinetics of bone formation via fluorescent labeling as a tool to detect new mineral apposition will be implemented. The present results are consistent with other studies demonstrating the positive action of HE on bone properties^22,86–88^. Although a bone-sparing effect on skeletal bone was observed, HE was associated with reduced weight gain, which is consistent with other literature^89^. Chen et al.^90^ suggested that the flavonoid corylin exerts anti-obesity effects through the browning of white adipocytes, activating brown adipose tissue and promoting lipid metabolism. Further studies on the effect of HE on weight, fat and metabolism is warranted in addition to further mechanistic investigations of the effects of HE in bone.

## Conclusion

In conclusion, our findings show, for the first time, that HE as a dietary supplement has a clear and promising modulatory role in BMP2-induced bone regeneration potentially via control of inflammation, effect on bone cells’ function, and collagen maturation and can lead to a positive control of BMP2-induced regeneration. This work supports the idea that HE may be a fast-track, implementable, cost-effective method of improving BMP2 application in current regenerative bone therapies due to its multiple positive effects on controlling inflammation, improving bone matrix quality and ensuring sufficient and adverse-event free bone healing. This study exemplifies the benefits of dietary supplementation in modulation of craniofacial and skeleton bone parameters improving oral and systemic health.

## Data Availability

All relevant data can be found within the article. Any raw data supporting reported results can be requested by contacting the corresponding author.
